# Electronic cigarette use and its association with asthma, chronic obstructive pulmonary disease (COPD) and asthma-COPD overlap syndrome among never cigarette smokers

**DOI:** 10.18332/tid/142579

**Published:** 2021-10-21

**Authors:** Emine Bircan, Ummugul Bezirhan, Austin Porter, Pebbles Fagan, Mohammed S. Orloff

**Affiliations:** 1 Department of Epidemiology, Fay W. Boozman College of Public Health, University of Arkansas for Medical Sciences, Little Rock, United States; 2 Teachers College, Columbia University, New York, United States; 3 Department of Health Policy and Management, Fay W. Boozman College of Public Health, University of Arkansas for Medical Sciences, Little Rock, United States; 4 Arkansas Department of Health, Little Rock, United States; 5 Department of Health Behavior and Health Education, Fay W. Boozman College of Public Health, University of Arkansas for Medical Sciences, Little Rock, United States; 6 Center for the Study of Tobacco, Department of Health Behavior and Health Education, Fay W. Boozman College of Public Health, University of Arkansas for Medical Sciences, Little Rock, United States; 7 Winthrop P. Rockefeller Cancer Institute, University of Arkansas for Medical Sciences, Little Rock, United States

**Keywords:** e-cigarettes, combustible cigarettes, asthma, COPD, Asthma-COPD Overlapping Syndrome (ACOS)

## Abstract

**INTRODUCTION:**

Although smoking is a strong risk factor for lung diseases including asthma, COPD, and asthma-COPD overlap syndrome (ACOS), studies are needed to examine the association between e-cigarettes and asthma, COPD, and ACOS. This study evaluated the association between e-cigarette use and self-reported diagnosis of asthma, COPD, and ACOS using a large nationally representative sample of adults aged ≥18 years in the United States.

**METHODS:**

Cross-sectional data from the Behavioral Risk Factor Surveillance System (BRFSS) from 2016 to 2018 were used to examine self-reported information on current e-cigarette use, demographic variables, and asthma and COPD status among never cigarette smokers (n=8736). Asthma and COPD were measured by self-reported diagnosis, and respondents who reported having both diagnoses were then classified as having ACOS. Of the 469077 never cigarette smokers, 4368 non-e-cigarette users were 1:1 propensity score-matched to e-cigarette users on age, sex, race/ethnicity and education level. We used multinomial logistic regression to examine association between current e-cigarette use and self-report asthma, COPD, and ACOS while controlling for marital status and employment in addition to matching variables.

**RESULTS:**

Compared with never e-cigarette users, e-cigarette users had increased odds of self-reported ACOS (OR=2.27; 95% CI: 2.23–2.31), asthma (OR=1.26; 95% CI: 1.25–1.27) and COPD (OR=1.44; 95% CI: 1.42–1.46).

**CONCLUSIONS:**

Our findings suggest that e-cigarette use is associated with an increased odds of self-reported asthma, COPD, and ACOS among never combustible cigarette smokers. BRFSS provides cross-sectional survey data, therefore a causal relationship between e-cigarette use and the three lung diseases cannot be evaluated. Future longitudinal studies are needed to validate these findings.

## INTRODUCTION

Overall tobacco use has declined in the United States over the past 50 years, but the prevalence of electronic cigarette (e-cigarette) use has increased rapidly in United States since their introduction in 2007^1^. E-cigarettes are battery-powered devices that create an aerosol by heating up liquid that usually includes nicotine, flavorings, propylene glycol, vegetable glycerin and other chemicals^2^. The tobacco industry introduced e-cigarettes as a safer alternative to tobacco smoking^3^. Around 8.1 million (3.2%) US adults reported using e-cigarettes in 2018; the prevalence of e-cigarette use increased from 2.8% to 3.2% during 2017–2018^4^.

The causal relationship between cigarette smoking and respiratory diseases is well documented^5^. Over 80% of chronic obstructive pulmonary disease (COPD) cases are caused by cigarette smoking and cigarette smoking exacerbates asthma in adults^5^. The respiratory health effects of e-cigarette use are still not clear. Several studies that have examined the negative short-term health effects of e-cigarette use found that people who had smoked e-cigarettes for several months experienced an increase in shortness of breath and coughs^2,6^. A recent consensus report from the National Academies of Science, Engineering, and Medicine on the Public Health Consequences of E-cigarettes^7^ showed moderate evidence that e-cigarettes increase coughing, wheezing as well as asthma exacerbation in adolescents. There is little evidence showing improvement in lung function and respiratory symptoms among adult smokers with asthma who switch to e-cigarettes completely or partly (dual use)^7^.

Data on the relationship between e-cigarette use and respiratory diseases such as COPD and asthma are scarce. Existing studies suggest that e-cigarette use is associated with chronic bronchitis, emphysema, COPD^8^ and asthma^9^. COPD and lifetime asthma are diagnosed in approximately 6% and 13% of the US adult population, respectively^10,11^. Although it varies by population, prevalence of asthma-COPD overlap syndrome (ACOS) has been estimated to be between 13% and 38%^12,13^. According to new guidelines, this overlap syndrome is characterized by persistent airflow limitation with several features of both asthma and COPD^14^. Characteristics of ACOS has not been fully understood due to challenges in defining its clinical features and differences in diagnosis^15^.

Many e-cigarette users also use cigarettes, making it difficult to untangle the unique contributions of e-cigarettes to COPD and asthma development. In addition, the long-term respiratory health effects of e-cigarette smoking on ACOS have not been thoroughly studied, due to challenges of clinical similarities between the two diseases^16^. Therefore, we studied the association between e-cigarette use and self-reported asthma, COPD, and ACOS among never combustible cigarette smokers using a large, nationally representative survey in the US to address this gap. We hypothesize that e-cigarette use is associated with chronic respiratory disorders including asthma, COPD and ACOS in adults.

## METHODS

### Population

**Figure 1:**
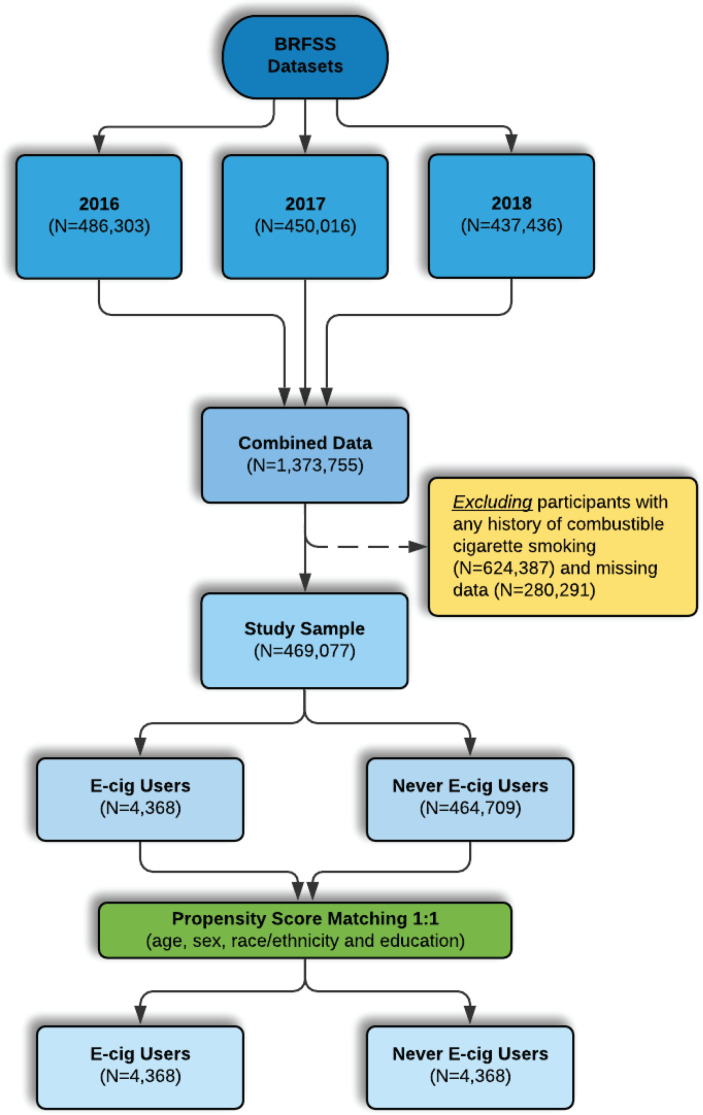
Flow chart for participants included in the study

We used 2016 (n=486303), 2017 (n=450016), and 2018 (n=437436) data from the Behavioral Risk Factor Surveillance System (BRFSS), a large cross-sectional telephone survey of non-institutionalized adults aged ≥18 years in the US^17^. The BRFSS gathers data on health risk behaviors, prevention practices, and access to healthcare as it relates to chronic disorders^18^. Our study population consisted of participants, aged ≥18 years, who were never smokers of conventional cigarettes. Participants were considered never smokers of conventional cigarettes if they answered ‘No’ to the question: ‘Have you smoked at least 100 cigarettes in your entire life?’. Therefore, we excluded current or former cigarette smokers (defined as lifetime smoking >100 cigarettes). Participants who did not respond to questions regarding respiratory disorders (asthma, COPD, ACOS) and e-cigarette use were excluded from the analysis (Figure 1). Additionally, participants who reported having used e-cigarettes in their lifetime and currently do not use e-cigarettes (defined as former e-cigarette users) were excluded from the study population because of lack of clarity of exposure status and residual effects that have resulted from the e-cigarette derived chemical exposure which contribute to changes in the continuum of the disease progression leading to molecular changes detectable by biomarker analysis rather than reports. These former e-cigarette users have used e-cigarettes sometime in their entire life and have quit for different reasons. Studies have shown that while cessation of tobacco use diminishes the risk of experiencing longterm adverse health effects, past history of tobacco use is still associated with increased risk of lung diseases compared to never having smoked^19^. Of 469077 never combustible cigarette smokers, there were 4368 e-cigarette users and 464709 never e-cigarette users. After propensity score matching (PSM), the study sample included 4368 e-cigarette users and 4368 never e-cigarette users matched on age, sex, race/ethnicity and education level.

### Measures

#### Current e-cigarette users and never e-cigarette users

Respondents who responded ‘Yes’ to the first question ‘Have you ever used an e-cigarette or other electronic vaping product, even just one time, in your entire life?’ were considered as ever e-cigarette users, and those who responded ‘No’ were considered as never e-cigarette users. Ever e-cigarette users were then asked: ‘Do you now use e-cigarettes or other electronic vaping products every day, some days, or not at all?’. Those who responded ‘every day’ or ‘some days’ were defined as current e-cigarette users. Our study focused on current e-cigarette users as those who qualify to be among the exposed. Note that those who responded no to 'every day' or 'some days' are participants who reported having used e-cigarettes in their lifetime and currently do not use e-cigarettes are classified as former e-cigarette users and were excluded from the study population because of lack of clarity of exposure status.

#### Asthma, COPD, and ACOS

Participants who responded ‘No’ to ‘Has a doctor, nurse, or other health professional ever told you that you have chronic obstructive pulmonary disease or COPD, emphysema, or chronic bronchitis?’ were defined as currently having a diagnosis of asthma if they further answered affirmatively to both ‘Has a doctor, nurse, or other health professional ever told you that you have asthma?’ and ‘Do you still have asthma?’. Similarly, respondents who reported to have no asthma were defined as having a diagnosis of COPD if they responded ‘Yes’ to ‘Has a doctor, nurse, or other health professional ever told you that you have COPD?'. People who responded ‘Yes’ to both asthma and COPD diagnosis were then classified as ACOS.

#### Sociodemographic and other risk factors

We examined sociodemographic and other risk factors as covariates. The survey included items on age in years (recoded as 5 years intervals from 18 to 80+), gender (female/male), race/ethnicity (non-Hispanic White, non-Hispanic Black, Hispanic, non-Hispanic Multiracial, other), educational level (below high school, graduated from high school, attended college or technical school, graduated from college or technical school), marital status (married, never married or member of unmarried couple, divorced or widowed or separated), income level (<15000, 15000–25000, 25000–35000, 35000–50000, >50000 US$), employment status (employed, homemaker or student, unemployed), body mass index (BMI) (underweight <18.5, normal weight 18.5–24.9, overweight 25–29.9, and obese >30 kg/m^2^), current physical activity or exercise in the last 30 days (Yes/No), health insurance coverage (Yes/No), and needing to see a doctor but not being able to afford it (Yes/No).

### Analysis

The 2016, 2017 and 2018 data were combined and analyzed according to the published Centers for Disease Control and Prevention recommendations, using suggested weighting methodology to improve the representativeness of data^20^. Never e-cigarette users were matched to e-cigarette users on age, sex, race/ethnicity and education using 1:1 propensity score matching to account for selection bias. Specifically, the nearest neighbor matching algorithm was implemented using the MatchIt package^21^. While there are various methods to employ propensity score matching, nearest neighbor matching is the most common technique that is used in both social sciences and medical literature^22^. This method utilizes a distance measure to quantify the closest match between treated and controlled units. First, propensity scores were estimated based on given covariates using logistic regression. Then, the matching is performed with 1:1 nearest neighbor pair matching without replacement using the difference between the propensity scores of each treated and control unit as a distance measure. For each individual in treatment group (e-cigarette user) a control unit (never e-cigarette user) was selected using the difference between their propensity score estimates. To assess the balance, standardized difference in mean (SDM) values were calculated. SDMs were smaller than 0.01 and variance ratios were 1 for all covariates that are used in matching. Potential confounders were identified by creating Directed Acyclic Graphs (DAGs) based on existing literature^23^. After matching is performed with age, sex and race covariates, the other confounding variables including education level, marital status, income, employment, BMI, and psychical activity, were controlled for in the analysis. Multinominal logistic regression was used to examine the association between e-cigarette use and asthma, COPD as well as ACOS. P-values <0.05 were considered significant. All analyses were conducted using R version 3.0.2^24^ utilizing survey procedures that account for complex sampling design of BRFSS.

## RESULTS

### Matched population

**Figure 2:**
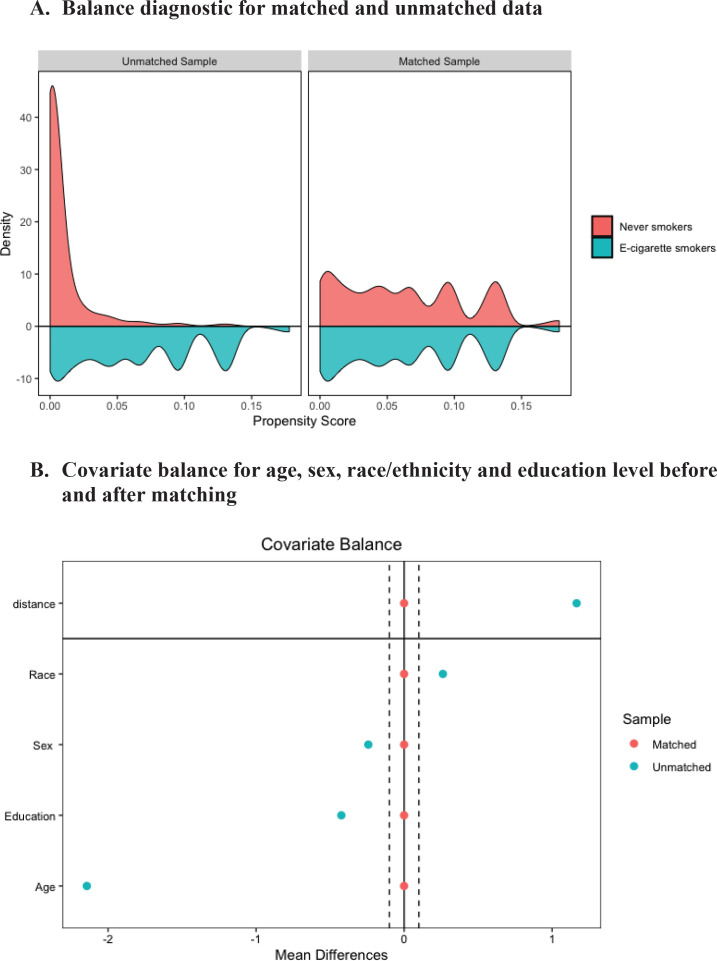
Balance diagnostic. A) Distribution balance for matched and unmatched data, B) Covariate balance for variable age, sex, race/ethnicity, and education before and after matching

The propensity score sample included 4368 e-cigarette users matched to 4368 never e-cigarette users. Distributional balance before and after PSM for variable age, sex, race/ethnicity and education level, respectively, is shown in Figure 2A. Density plot shows the distributional balance for age, sex, race/ethnicity, and education level before and after the matching. Figure 2B shows covariate balance measured by standardized mean difference indicating standardized difference in means before and after matching on age, sex, and race/ethnicity. For a given variable when standardized difference in mean is between the threshold value as indicated by two dashed vertical lines, the balance is considered to be achieved.

Table 1 shows the population weighted baseline distribution of groups after 1:1 propensity score matching on age, sex, race/ethnicity and education level. In both groups, the age of respondents was 18–24 years. Both groups consisted of a greater proportion non-Hispanic White (64.1%), male (65.6%), and reported to have attended college or technical school (35.9%).

**Table 1:** Baseline characteristics of study population after matching on age, sex, race/ethnicity and education level, BRFSS 2016–2018, US (N=8736)

*Characteristics*	*E-cigarette use status ^a^*		*p*
	*E-cigarette users (N=4368) n (%)*	*Never e-cigarette users (N=4368) n (%)*	
**Median age range** (years)	18–24	18–24	-
**Women**	1504 (34.40)	1504 (34.40)	-
**Race**			-
White non-Hispanic	2798 (64.10)	2798 (64.10)	
Black non-Hispanic	404 (9.20)	404 (9.20)	
Other race non-Hispanic	345 (7.90)	345 (7.90)	
Multiracial non-Hispanic	190 (4.30)	190 (4.30)	
Hispanic	631 (14.40)	631 (14.40)	
**Education level**			-
Below high school	243 (5.60)	243 (5.60)	
Graduated from high school	1507 (34.50)	1507 (34.50)	
Attended college or technical school	1567 (35.90)	1567 (35.90)	
Graduated from college or technical school	1051 (24.10)	1051 (24.10)	
**Marital status**			<0.001
Married	853 (19.50)	1354 (30.70)	
Never married/cohabiting	3141 (71.90)	2760 (63.20)	
Divorced/widowed/separated	374 (8.60)	267 (6.10)	
**Income** (US$)			0.726
<15000	438 (10.00)	447 (10.20)	
15000–25000	819 (18.80)	780 (17.90)	
25000–35000	518 (11.90)	498 (11.40)	
35000–50000	665 (15.20)	666 (15.20)	
>50000	1928 (44.10)	1977 (45.30)	
**Employment**			<0.001
Employed	2876 (65.80)	2817 (64.50)	
Homemaker/student	965 (22.10)	1001 (25.20)	
Unemployed	527 (12.10)	450 (10.30)	
**BMI** (kg/m^2^)			0.424
Underweight (<18.5)	140 (3.20)	122 (2.80)	
Normal weight (18.5–24.9)	1852 (42.40)	1832 (41.90)	
Overweight (25.0–29.9)	1327 (30.40)	1311 (30.00)	
Obese (>30.0)	1049 (24.00)	1103 (25.30)	
**Physical activity**			0.104
Performs physical activity or exercise	3690 (84.50)	3633 (83.20)	
No physical activity or exercise in last 30 days	678 (15.50)	735 (16.80)	
**Pulmonary disease**			<0.001
Asthma	430 (9.80)	314 (7.20)	
COPD	90 (2.10)	58 (1.30)	
ACOS	66 (1.50)	31 (0.70)	

a Former e-cigarette users who reported to having used e-cigarettes in their lifetime and currently do not use e-cigarettes were excluded from the study population.

After matching, there were only significant differences with marital status, employment and pulmonary diseases. A greater proportion of never e-cigarette users were married (31.0%) compared to e-cigarette users (19.5%). Employment for e-cigarette users and never e-cigarette users was 65.8% and 66.3%, respectively, with a greater proportion of never e-cigarette users reporting an income >50000 US$ (44.1% vs 46.2%). A greater proportion of e-cigarette users reported having pulmonary diseases compared with never e-cigarette users. Approximately 9.8% of e-cigarette users were diagnosed with asthma compared with 7.2% among never e-cigarette users. Also, the proportion of COPD and ACOS was greater among e-cigarette users (2.1% vs 1.3%) and (1.5% vs 0.7%), respectively. For all other variables, the differences between the two groups were not significantly different.

**Figure 3:**
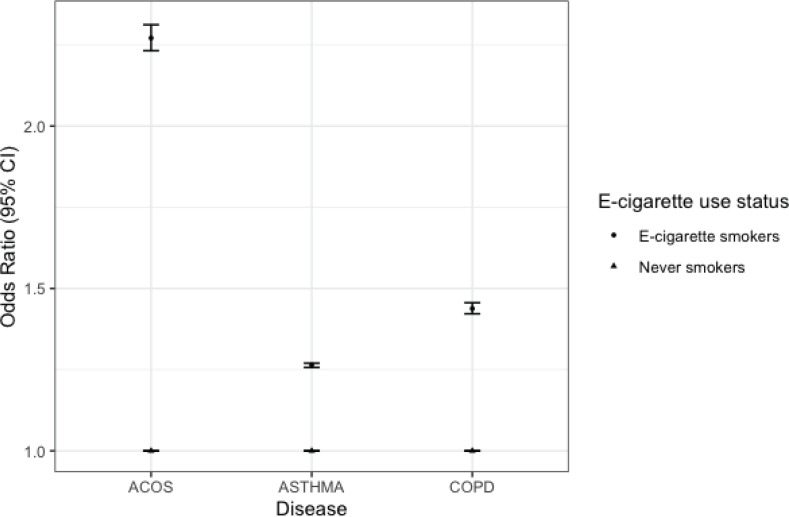
Multinomial regression of the association between e-cigarette use and asthma, COPD, and ACOS among adults aged ≥18 years BRFSS, 2016-2018

Figure 3 shows estimates from multinomial regression analysis after removing participants with missing variables and matching never e-cigarette users to e-cigarette users on age, sex, race/ethnicity and education level. After adjusting for confounding variables, e-cigarette users had increased odds of self-reported ACOS (OR=2.27; 95% CI: 2.23–2.31), asthma (OR=1.26, 95% CI: 1.25–1.27) and COPD ( OR=1.44; 95% CI : 1.42–1.46).

## DISCUSSION

To our knowledge, this is the first study to examine the association between e-cigarette use and self-reported asthma, COPD, and ACOS among never combustible cigarette smokers using a large nationally representative survey of the US population, i.e. BRFSS. Our findings show that e-cigarette use among adults is associated with chronic respiratory disorders such as asthma, COPD, and ACOS. Using our matched design (i.e. age, sex, race/ethnicity, and education level). The odds of ACOS were twice as high among e-cigarette users compared to never e-cigarette users. Data from this large nationally representative sample also showed that e-cigarette users had significantly greater odds of asthma and COPD. The findings from this study suggest the need to further investigate the long-term and short-term health effects since the age of those at risk in our matched design was 18–24 years.

Consistent with other national studies, the majority of adult e-cigarette users were aged 18–24 years^25,26^. This is not surprising because national studies consistently show higher e-cigarette use among young adults compared to older adults^27,28^. However, what is unique is that there are adults within these age group reporting COPD and ACOS. Although asthma can present in younger individuals, including children^29^, onset of COPD commonly occurs in those over the age of 40 years^30^. A large international study has recently estimated the prevalence of COPD according to the GOLD standard among young adult population (aged 20–44 years)^31^. The results from this study support our findings that early onset of COPD-like features likely develop earlier than is usually believed and can inform early detection of the disease and preventive measures. As expected with this young age group, a small percentage of subjects had mild to moderately severe COPD symptoms and none from this age group was found in the most severe stage of COPD (i.e. stage IV). This paves the way for biomarkers such as alpha1antitrypsin deficiency to identify younger people who are likely to develop early stages of COPD^32^.

### Strengths and limitations

A major strength of our study is the large, representative sample of the US population to test our hypothesis. By selecting never cigarette smokers, we removed the confounding effect attributed to dual use of conventional cigarettes. We also used propensity score matching to remove confounding effects of potential confounders including age, sex, race/ethnicity and education level.

Additionally, former e-cigarette users who reported to having used e-cigarettes in their lifetime and currently do not use e-cigarettes were excluded from the study population. Accumulation of tobacco-specific carcinogens or volatile organic compounds^33,34^ due to previous e-cigarette use or conventional smoking can contribute to pre-clinical changes of lung disorders. Additional studies have shown that several exposure biomarkers of effect that are related to nicotine inhalation, such as leukocyte count^19,35-37^, have shown levels that remain increased in former smokers compared to never smokers^19^. Therefore, excluding 'former e-cigarette-users' from being part of the mix of the comparison or reference group in our study will minimize misclassification that can bias the effect measure estimate (i.e. OR) away from the null.

We acknowledge that this is a cross-sectional study design, as such, attempts to precisely identify individuals who are truly exposed or not exposed (i.e. e-cigarette users and never e-cigarette users) in the absence of biomarkers are highly warranted to minimize introducing bias.

Our study is not without limitations. We cannot completely discount residual confounding due to unknown factors. Considering that the BRFSS is a cross-sectional survey design, a causal relationship between e-cigarette use and asthma, COPD, and ACOS cannot be evaluated. Particularly, considering that the age of the study respondents who are e-cigarette users was 18–24 years and that asthma prevalence is higher mostly during childhood^38^, it is possible that some e-cigarette users may have started after asthma diagnosis, ruling out causality. Frequency of self-reported disease outcomes were low in both e-cigarette users and matched never e-cigarette users. Therefore, it should be noted that the statistically significant difference and more precise odds ratio estimates are observed when the three disease symptoms are combined. We acknowledge that exposure and outcome were self-reported, and there are no data on e-cigarette use initiation, duration, intensity (puffs/day) as well as specific flavor used. Also, we were not able to verify subjects in the two groups using specific biomarkers of exposure. Although we did not imply a causal association, chronic inhalation of e-cigarette vapor that contains nicotine has been shown to disrupt airway barrier function and induce systemic inflammation in mice^39^. Self-reported diagnosis of asthma by a healthcare provider is commonly used by government agencies and scientists in general, as it has been shown to correlate well with the diagnosis of asthma^40^. However, self-reported physician diagnosis of COPD may have high specificity and low sensitivity^41^ and hence would bias the effect measure estimate towards the null but this does not explain our finding. Additionally, respiratory disorders are complex disorders resulting from many factors, which may include the interaction between genetic and environmental factors^42^. In our study, we were not able to account for gene-environment interaction. Despite these limitations, combined data of 2016, 2017, and 2018 from BRFSS, a large nationally representative US sample provided us with a large sample size to study e-cigarette use specifically among this unique population of never combustible cigarette users.

## CONCLUSIONS

Data from this large nationally representative sample suggest that e-cigarette use is associated with increased odds of self-reported asthma, COPD, and ACOS among never combustible cigarette smokers. The odds of ACOS were twice as high among e-cigarette users compared with never e-cigarette users of conventional cigarettes. The findings from this study suggest the need to further investigate the long-term and short-term health effects of e-cigarette use, since the age of those at risk in our study was 18–24 years. This is not surprising because national studies consistently show higher e-cigarette use among young adults compared to older adults.

## References

[1] Noel JK, Rees VW, Connolly GN (2011). Electronic cigarettes: a new ‘tobacco’industry?. Tob Control.

[2] Grana R, Benowitz N, Glantz SA (2014). E-cigarettes: a scientific review. Circulation.

[3] Goniewicz ML, Smith DM, Edwards KC (2018). Comparison of nicotine and toxicant exposure in users of electronic cigarettes and combustible cigarettes. JAMA Netw Open.

[4] Creamer MR, Wang TW, Babb S (2019). Tobacco product use and cessation indicators among adults—United States, 2018. MMWR Morb Mortal Wkly Rep.

[5] National Center for Chronic Disease Prevention and Health Promotion US - Office on Smoking and Health (2014). The health consequences of smoking—50 years of progress: a report of the Surgeon General.

[6] Scheffler S, Dieken H, Krischenowski O, Förster C, Branscheid D, Aufderheide M (2015). Evaluation of E-cigarette liquid vapor and mainstream cigarette smoke after direct exposure of primary human bronchial epithelial cells. Int J Environ Res Public Health.

[7] National Academies of Sciences, Engineering, and Medicine, Health and Medicine Division, Board on Population Health and Public Health Practice, Committee on the Review of the Health Effects of Electronic Nicotine Delivery Systems (2018). Public health consequences of e-cigarettes.

[8] Wills TA, Pagano I, Williams RJ, Tam EK (2019). E-cigarette use and respiratory disorder in an adult sample. Drug Alcohol Depend.

[9] Osei AD, Mirbolouk M, Orimoloye OA (2019). The association between e-cigarette use and asthma among never combustible cigarette smokers: behavioral risk factor surveillance system (BRFSS) 2016 & 2017. BMC Pulm Med.

[10] American Lung Association Asthma Trends and Burden.

[11] Ford ES, Croft JB, Mannino DM, Wheaton AG, Zhang X, Giles WH (2013). COPD surveillance—United States, 19992011. Chest.

[12] Barrecheguren M, Esquinas C, Miravitlles M (2015). The asthma–chronic obstructive pulmonary disease overlap syndrome (ACOS): opportunities and challenges. Curr Opin Pulm Med.

[13] Harada T, Yamasaki A, Fukushima T (2015). Causes of death in patients with asthma and asthma–chronic obstructive pulmonary disease overlap syndrome. Int J Chron Obstruct Pulmon Dis.

[14] Gibson P, Simpson J (2009). The overlap syndrome of asthma and COPD: what are its features and how important is it?. Thorax.

[15] Bateman ED, Reddel HK, van Zyl-Smit RN, Agusti A (2015). The asthma–COPD overlap syndrome: towards a revised taxonomy of chronic airways diseases?. Lancet Respir Med.

[16] Hines KL, Peebles RS (2017). Management of the asthmaCOPD overlap syndrome (ACOS): a review of the evidence. Curr Allergy Asthma Rep.

[17] Centers for Disease Control and Prevention Behavioral Risk Factor Surveillance System: 2016 Summary Data Quality Report.

[18] Centers for Disease Control and Prevention (2014). About BRFSS.

[19] Parry H, Cohen S, Schlarb JE (1997). Smoking, alcohol consumption, and leukocyte counts. Am J Clin Pathol.

[20] Centers for Disease Control Prevention Behavioral Risk Factor Surveillance System 2016. Weighting BRFSS data: BRFSS 2016.

[21] Ho D, Imai K, King G, Stuart E, Whitworth A MatchIt: Nonparametric Preprocessing for Parametric Causal Inference [computer program].

[22] Austin PC (2010). The performance of different propensity-score methods for estimating differences in proportions (risk differences or absolute risk reductions) in observational studies. Stat Med.

[23] Greenland S, Pearl J, Robins JM (1999). Causal diagrams for epidemiologic research. Epidemiology.

[24] R Core Team (2013). R: A language and environment for statistical computing.

[25] Schoenborn CA, Gindi RM (2015). Electronic cigarette use among adults: United States, 2014. NCHS Data Brief.

[26] Dai H, Leventhal AM (2019). Prevalence of e-cigarette use among adults in the United States, 2014-2018. JAMA.

[27] Villarroel MA, Cha AE, Vahratian A (2020). Electronic cigarette use among US adults, 2018. NCHS Data Brief.

[28] Mayer M, Reyes-Guzman C, Grana R, Choi K, Freedman ND (2020). Demographic Characteristics, Cigarette Smoking, and e-Cigarette Use Among US Adults. JAMA Netw Open.

[29] Ebmeier S, Thayabaran D, Braithwaite I, Bénamara C, Weatherall M, Beasley R (2017). Trends in international asthma mortality: analysis of data from the WHO Mortality Database from 46 countries (1993–2012). Lancet.

[30] Wheaton AG, Cunningham TJ, Ford ES, Croft JB (2015). Employment and activity limitations among adults with chronic obstructive pulmonary disease—United States, 2013. MMWR Morb Mortal Wkly Rep.

[31] De Marco R, Accordini S, Cerveri I (2004). An international survey of chronic obstructive pulmonary disease in young adults according to GOLD stages. Thorax.

[32] Rahaghi FF, Sandhaus RA, Strange C (2012). The prevalence of alpha-1 antitrypsin deficiency among patients found to have airflow obstruction. COPD.

[33] Whiting DR, Guariguata L, Weil C, Shaw J (2011). IDF diabetes atlas: global estimates of the prevalence of diabetes for 2011 and 2030. Diabetes Res Clin Pract.

[34] Murphy SE, Park SL, Balbo S (2018). Tobacco biomarkers and genetic/epigenetic analysis to investigate ethnic/ racial differences in lung cancer risk among smokers. NPJ Precis Oncol.

[35] Phillips AN, Neaton JD, Cook DG, Grimm RH, Shaper AG (1992). The leukocyte count and risk of lung cancer. Cancer.

[36] Sunyer J, Muñoz A, Peng Y (1996). Longitudinal relation between smoking and white blood cells. Am J Epidemiol.

[37] Wald NJ, Thompson SG, Law MR, Densem JW, Bailey A (1989). Serum cholesterol and subsequent risk of cancer: results from the BUPA study. Br J Cancer.

[38] Moorman JE, Akinbami LJ, Bailey C (2012). National surveillance of asthma: United States, 2001-2010. Vital Health Stat 3.

[39] Crotty Alexander LE, Drummond CA, Hepokoski M (2018). Chronic inhalation of e-cigarette vapor containing nicotine disrupts airway barrier function and induces systemic inflammation and multiorgan fibrosis in mice. Am J Physiol Regul Integr Comp Physiol.

[40] Mirabelli MC, Beavers SF, Flanders WD, Chatterjee AB (2014). Reliability in reporting asthma history and age at asthma onset. J Asthma.

[41] Murgia N, Brisman J, Claesson A, Muzi G, Olin AC, Torén K (2014). Validity of a questionnaire-based diagnosis of chronic obstructive pulmonary disease in a general population-based study. BMC Pulm Med.

[42] Kleeberger SR, Peden D (2005). Gene-environment interactions in asthma and other respiratory diseases. Annu Rev Med.

