# Electronic cigarette use and its association with asthma, chronic obstructive pulmonary disease (COPD) and asthma-COPD overlap syndrome among never cigarette smokers

**DOI:** 10.18332/tid/141989

**Published:** 2021-10-21

**Authors:** Emine Bircan, Ummugul Bezirhan, Austin Porter, Pebbles Fagan, Mohammed S. Orloff

**Affiliations:** 1 Department of Epidemiology, Fay W. Boozman College of Public Health, University of Arkansas for Medical Sciences, Little Rock, United States; 2 Teachers College, Columbia University, New York, United States; 3 Department of Health Policy and Management, Fay W. Boozman College of Public Health, University of Arkansas for Medical Sciences, Little Rock, United States; 4 Arkansas Department of Health, Little Rock, United States; 5 Department of Health Behavior and Health Education, Fay W. Boozman College of Public Health, University of Arkansas for Medical Sciences, Little Rock, United States; 6 Center for the Study of Tobacco, Department of Health Behavior and Health Education, Fay W. Boozman College of Public Health, University of Arkansas for Medical Sciences, Little Rock, United States; 7 Winthrop P. Rockefeller Cancer Institute, University of Arkansas for Medical Sciences, Little Rock, United States

The authors would like to express their apologies and regret for the errors in the original published version of the abovementioned article. The corrected article follows.

DOIs

Original Article: https://doi.org/10.18332/tid/132833

Correction: https://doi.org/10.18332/tid/141989

Corrected Article: https://doi.org/10.18332/tid/142579

